# Retinal Function Deficits in American Staffordshire Terriers with a Late-Onset Neurodegenerative Disease Associated with an *ARSG* Variant

**DOI:** 10.3390/vetsci12111078

**Published:** 2025-11-12

**Authors:** Grace R. Kick, Samantha L. Marzano, Juri Ota-Kuroki, Garrett Bullock, Martin L. Katz

**Affiliations:** 1 Department of Ophthalmology, School of Medicine, University of Missouri, Columbia, MO 65212, USA; grace.robinson@health.missouri.edu (G.R.K.); samanthamarzanodvm@gmail.com (S.L.M.); 2 Department of Veterinary Medicine and Surgery, College of Veterinary Medicine, University of Missouri, Columbia, MO 65211, USA; otaj@missouri.edu; 3 Canine Genetics Laboratory, College of Veterinary Medicine, University of Missouri, Columbia, MO 65212, USA; gebkd2@missouri.edu

**Keywords:** electroretinogram, Usher type IV, arylsulfatase G, lysosomal storage disorder, retinal pigment epithelium

## Abstract

American Staffordshire Terriers (ASTs) with a c.296G>A variant in *ARSG* develop progressive ataxia, cerebellar atrophy, and neuronal accumulation of autofluorescent storage material. Human subjects with *ARSG* variants exhibit hearing loss and rod–cone dystrophy without apparent other neurological involvement and *arsg* knockout mice exhibit progressive ataxia, lysosomal storage, and photoreceptor loss. Owners of 8 of 11 affected ASTs evaluated for the *ARSG* risk variant reported observing visual impairment in their dogs, suggesting that the canine disease may involve retinal dysfunction consistent with human subjects and mice with *ARSG* variants. To assess whether this might be the case, electroretinography was performed on four affected and three unaffected ASTs. Three affected dogs that were exhibiting signs of ataxia had attenuated electroretinogram (ERG) amplitudes indicative of rod and cone photoreceptor dysfunction, while ERG responses were not attenuated in a younger dog that had not yet shown signs of ataxia or visual impairment. Autofluorescent inclusions were observed in the retinal pigment epithelium and retinal ganglion cell layer of two affected dogs that were euthanized due to neurological disease progression. The results from these cases indicate that standardized electroretinography can be used to detect retinal dysfunction in dogs with the *ARSG*-related disorder and in other disorders in which dogs exhibit apparent impairment in visually mediated behavior.

## Simple Summary

Humans, mice, and dogs with mutations in the arylsulfatase G gene develop diseases with differing symptoms. Affected human subjects suffer hearing and vision loss only. Mice with a null mutation develop progressive ataxia as well as retinal degeneration. Like mice lacking a functional copy of this gene, American Staffordshire Terriers (ASTs) with an arylsulfatase G gene mutation suffer from progressive ataxia, but affected dogs have not been evaluated for the type of visual impairment that affects human patients and mice. Affected ASTs whose DNA samples were submitted for genetic testing were reported by their owners to exhibit impaired vision. To assess whether this was related to impaired retinal function, objective measurement of retinal responses to light stimuli was performed on affected and unaffected ASTs. The response amplitudes were consistently lower in the affected dogs, reflecting impaired retinal function. These results suggest that while some disease signs vary between humans, mice, and dogs with arylsulfatase G gene mutations, visual impairment is a consistent feature in all three species. Confirmation of this finding will require assessing retinal function in additional dogs with the disorder.

## 1. Introduction

American Staffordshire Terriers (ASTs) homozygous for a c.296G>A substitution (p.R99H) in the *ARSG* gene and *arsg* knockout mice display reduced arylsulfatase G (ARSG) enzyme activity and develop a late-onset neurodegenerative condition clinically characterized primarily by slowly progressive ataxia [1,2]. ARSG-deficient ASTs and mice exhibit cerebellar atrophy, loss of Purkinje cells, and accumulation of autofluorescent and periodic acid–Schiff-positive storage material in Purkinje cells and other cells in the brain [1,2]. It was proposed that the canine AST disorder be classified as a form of neuronal ceroid lipofuscinosis (NCL), a group of rare inherited lysosomal storage disorders characterized by intracellular accumulation of autofluorescent storage material and progressive neurodegeneration [1]. *arsg* knockout mice were found to have significant accumulation of glycosaminoglycans and the wildtype ARSG protein was shown to function as a lysosomal sulfatase, consistent with an alternative classification of the *ARSG* disorders as mucopolysaccharidoses (MPS) [2]. Retinal degeneration is a common feature of many lysosomal storage disorders, including most forms of NCL and MPS [3,4,5,6,7,8,9,10]. In a previous study, visual impairment was not noted in affected ASTs and no retinal pathology was reported [1]; however, in ARSG-deficient mice there was an early, progressive loss of photoreceptors [11].

Usher syndrome is a group of autosomal recessive diseases characterized by sensorineural hearing loss and rod–cone dystrophy. Mutations in genes with roles in inner ear hair cell development and photoreceptor maintenance underlie classic Usher syndrome; however, cases of atypical Usher syndrome have also been described with mutations in genes with less clear associations with these functions [12,13,14,15,16]. There have been 31 reported cases of atypical Usher syndrome associated with *ARSG* variants [17]. Most of these patients display hearing loss and rod–cone dystrophy. In contrast to ASTs and mice with *ARSG* mutations, human patients generally do not exhibit neurological involvement, with the exception of two patients reported to have mild cerebellar atrophy without ataxia [17,18]. Interestingly, one patient was recently reported to have the homozygous p.R99H variant homologous to that in affected ASTs and another to have one copy of the p.R99H variant, the other being a splice variant predicted to lead to a premature stop codon [17]. Neither patient exhibited any neurologic signs as of age 74 (homozygous patient) and age 55 (heterozygous patient). The homozygous p.R99H patient reported night blindness and photophobia starting in his early 40s and progressive hearing loss starting in his 50s. At the age of 55, the heterozygous p.R99H patient had rod–cone dystrophy with nondetectable scotopic and residual photopic responses and denied hearing loss.

Because visual impairment and retinal degeneration are associated with *ARSG* variants in mice and human subjects, a study was undertaken to determine whether retinal function was also affected in dogs homozygous for the *ARSG* disease variant.

## 2. Case Series

Dogs included in this study consisted of American Staffordshire Terriers (ASTs) whose owners had submitted DNA samples (blood or buccal swabs) for genetic screening to the University of Missouri Canine Genetics Laboratory and completed a standard neurological diseases questionnaire (see Supplemental File S1) on their dogs. Clients submitting samples for DNA testing agree to allow deidentified data from their dogs to be used for research. Dogs included in the study were at least two years of age and had been genotyped for the *ARSG* c.296G>A variant. The owners of these dogs were asked whether they would agree to enroll their dogs in a study that would entail an examination by a veterinary ophthalmologist that could include electroretinography. For dogs whose owners provided informed consent to have their dogs participate in this phase of the study, arrangements were made to have the dogs examined by a local veterinary ophthalmologist. If feasible, dogs underwent ophthalmological evaluation at the University of Missouri. Some dogs had to be excluded from the ophthalmological exam portion of the study if no local ophthalmologist could be found who could perform the examinations. Retinal function was assessed in a subset of dogs for which it was possible to obtain electroretinogram (ERG) recordings using a standardized protocol. A total of 11 ASTs homozygous for the risk allele and 18 ASTs homozygous or heterozygous for the reference allele which were at least 6 years of age were included in the study. Eyes were obtained with informed consent from the owners from two ASTs homozygous for the *ARSG* risk allele who did not undergo assessment of retinal function but who were euthanized due to progressive ataxia.

All of the ASTs that were homozygous for the *ARSG* risk variant that were 6 years or older exhibited late onset progressive ataxia, whereas one that was 4 years old at the time of assessment did not exhibit this disease sign. Owners of dogs homozygous for the *ARSG* reference allele reported no neurologic signs. Six ASTs underwent ophthalmological examinations at the University of Missouri Veterinary Health Center by a board-certified veterinary ophthalmologist. The examinations included retinal imaging and electroretinography. Dog ARSG-2 was examined by a board-certified veterinary ophthalmologist outside of the University of Missouri using the same protocol as the dogs examined in-house and the results were sent to us. For most of the dogs evaluated by ophthalmologists, fundus images were acquired and sent to the University of Missouri for evaluation by a board-certified veterinary ophthalmologist (JO). See Table 1 for the case demographics.

In addition to progressive ataxia, owners of dogs homozygous for the *ARSG* risk variant reported a number of other behavioral signs with onset that coincided with the onset of ataxia and that also progressed over a period of 4 or more years. Among these signs were decreased interest in food, anxiety, aggressiveness toward other dogs and people, increased sensitivity to loud sounds, tremors including head bobbing, decreased tolerance to grooming or bathing, compulsive circling and other compulsive behaviors, and a roached back posture. In an 8-year-old dog the ataxia had become so severe that the dog was no longer able to stand or ambulate without assistance (Video S1).

Experiments were performed in accordance with the University guidelines for clinical studies and protocols were approved by the University of Missouri Animal Care and Use Committee (protocol number 44562, approved 8 January 2025). Informed consent was obtained from the owners of the dogs that participated in this study. The studies were performed in accordance with the ARVO Statement for the Use of Animals in Ophthalmic and Vision Research.

## 3. Genotyping

The *ARSG* genotypes were determined by the Canine Genetics Laboratory at the University of Missouri College of Veterinary Medicine using an allelic discrimination assay. A custom TaqMan SNP genotyping assay (Applied Biosystems) was designed for genotyping individual dogs for the *ARSG* variant. The PCR primer sequences were 5′-GGCCTCCCTGCTCACC-3′ and 5′-GAGGTGACCGCAAAGTTGTG-3′. The competing probe sequences were 5′-VIC- TGGGCCTCCGCAACG-NFQ-3′ (reference allele) and 5′-FAM- TGGGCCTCCACAACG-NFQ-3′ (variant allele). The amplifications were conducted in 20 µL volumes with a TaqMan genotyping master mix (Applied Biosystems, Waltham, MA, USA) and included an initial denaturation at 95 °C for 10 min, followed by 40 cycles of denaturation at 95 °C for 15 s, primer annealing at 60 °C for 1 min, and a final extension at 60 °C for 30 s on a StepOnePlus real-time PCR system (Applied Biosystems). The genotypes of the cases evaluated in this study are indicated in Table 1.

## 4. Questionnaire

Owners of ASTs who submitted samples from their dogs to the Canine Genetics Laboratory for testing filled out a standardized neurologic disease questionnaire in which they were asked to describe behaviors and physical activities in 29 specific categories as normal, or mildly, moderately, or severely abnormal (Supplemental File S1). Questions relevant to this study included: difficulty in movement or coordination (ataxia), ability to see during the day (photopic vision), and ability to see at night in dim light (scotopic vision). The owners were prompted to elaborate on any abnormalities they observed in each of the parameters evaluated.

Owners of the majority of the genetically affected dogs reported visual impairment under either bright or dim lighting conditions or both in their affected dogs (Table 1), with ages of onset similar to the ages of onset of neurological signs. Photopic visually mediated behavior was reported by owners as normal in 7/11 dogs and mildly altered in 4/11. Scotopic visually mediated behavior was reported as normal in 3/11, mildly altered in 3/11, moderately altered in 4/11, and severely altered in 1/11. The latter dog was reported to have developed such severe visual impairment under dim light conditions as the disease progressed that the dog would not enter a dark room until a light was turned on. No signs of visual impairment were reported by owners of any of the dogs homozygous or heterozygous for the reference *ARSG* allele. Based on owner assessments of visually mediated behavior, dogs were classified as ARSG affected/normal vision, ARSG affected/impaired vision, ARSG normal/normal vision, and ARSG normal/impaired vision. Dogs were classified as vision impaired if the owners indicated any apparent visual deficits on the neurological diseases questionnaire. Chi-square analysis was performed to test the hypothesis that ARSG-affected dogs were more likely to exhibit behavior interpreted by their owners as reflecting visual impairment. Owners of dogs homozygous for the *ARSG* risk variant were significantly more likely to report that their dogs were visually impaired than owners of dogs homozygous or heterozygous for the reference *ARSG* allele (chi-square = 16.4, *p* = 0.00005, *n* = 29, df = 1).

## 5. Ophthalmic Examinations

Dogs in the study were screened prior to inclusion in the study to ensure no confounding ophthalmic abnormalities such as glaucoma, cataracts, or traumatic eye injuries. Each dog received a complete bilateral ophthalmic examination by a board-certified veterinary ophthalmologist using slit-lamp biomicroscopy of the adnexa and anterior segment as well as indirect ophthalmoscopy of the posterior segment (retina and optic nerve head). For cases in which the examining ophthalmologist had a fundus camera available, fundus images were acquired. Each examining ophthalmologist provided their assessment of retinal integrity based on the fundus examinations. In addition, all fundus images were reviewed by the same veterinary ophthalmologist (JO) for consistency in interpretation. Ophthalmic assessments were performed at the ages indicated in Table 1. None of the dogs evaluated for this study exhibited any apparent structural ocular abnormalities. Pupillary light reflexes were present for all dogs, intraocular pressures were normal, and no obvious retinal lesions were observed consistently in the affected dogs with fundus imaging.

## 6. Electroretinography

For those dogs for which it was feasible to perform a standardized ERG assessments, unilateral electroretinography was performed on the left eye to assess scotopic and photopic retinal responses [19]. Pupils were dilated with one drop of 1% tropicamide ophthalmic solution. Subdermal ground and reference needle electrodes were inserted under the skin over the rostral aspect of the occipital protuberance and halfway between the lateral canthus of the left eye and the base of the left ear, respectively. ERG-Jet gold-ring contact lens electrodes (Fabrinal SA, La Chaux-de-Fonds, Switzerland) were placed on the cornea with methylcellulose. Recordings were performed in the dark with a full-field flash HMsERG unit following the ‘Dog Diagnostic Protocol’ recommended by the European College of Veterinary Ophthalmologists (ECVO) [20]. The ERG instrumentation was calibrated by the manufacturer (OcuScience, Kansas City, MO, USA) within 6 months prior to data acquisition. Pure rod responses were elicited with five sets of ten 10.2 log photons/cm^2^/s (10 mcd/m^2^) flashes at 2 s intervals repeated four times over a 20 min period of dark adaptation (scotopic dim flashes); amplitudes from the fifth set, collected after 20 min of dark adaptation, are reported. Mixed rod and cone responses were elicited with four 12.65 photons/cm^2^/s (3 cd/m^2^) flashes at 10 s intervals (scotopic bright flash) and, 30 s later, four 13.2 log photons/cm^2^/s (10 cd/m^2^) flashes at 20 s intervals (scotopic high-intensity flash). Ganzfeld dome lights were then turned on (13.65 log photons/cm^2^/s, 30 cd/m^2^) and held over the dog’s eye for 10 min for light adaptation. Pure cone responses were elicited with 12.65 log photons/cm^2^/s (3 cd/m^2^) flashes at 0.5 s intervals (photopic single flash) and, 2 s later, a 3 cd/m^2^, 30 Hz flicker (photopic flicker). The average response of each set of flashes was used for analysis. For some of the dogs enrolled in the study, it was not possible for the examining ophthalmologists to perform ERG assessments according to the standard ECVO protocol, so ERG data on these dogs are not included. Each of the participating ophthalmologists were provided with the information on performing ERG assessments using the standard protocol. In most cases, the ophthalmologists did not have the appropriate ERG equipment or the time to obtain the ERG data according to the ECVO protocol. For some of these cases, ERG data were acquired, but due to differences in protocols and instrumentation the data could not be compared to data obtained using the standard protocol.

It has been established that for healthy dogs peak scotopic response amplitudes are reached after 20 min of dark adaptation [20]. To determine whether the *ARSG* variant affected the rate of dark adaptation, ERG flash response b-wave amplitudes were assessed at 5 min intervals after the onset of dark adaptation. While all three control dogs did not exhibit peak response amplitudes until after 20 min of dark adaptation, (lower) peak response amplitudes in the affected dogs all occurred after 10 to 15 min of dark adaptation.

**Figure 1:**
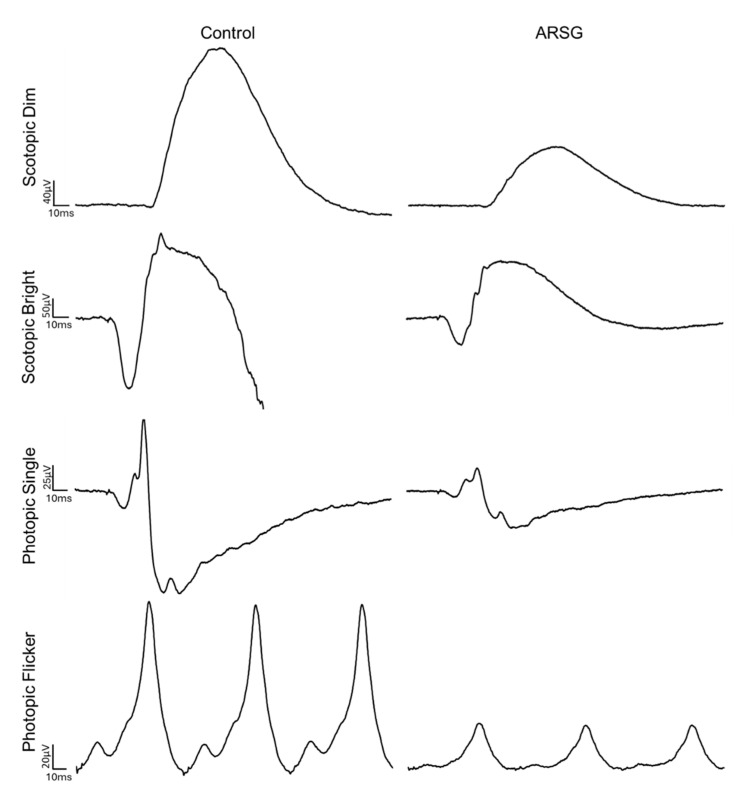
Example ERG responses from dogs Control-1 and ARSG-1.

**Figure 2:**
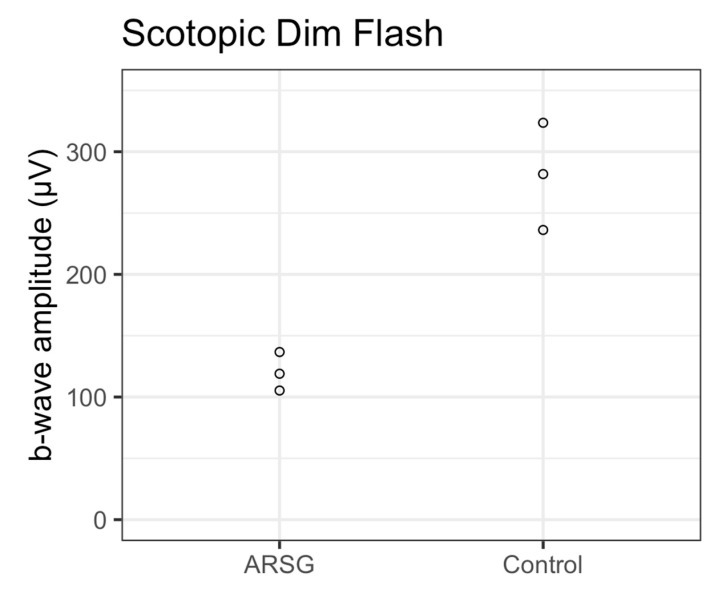
Rod photoreceptor responses as assessed by scotopic dim flash b-wave amplitudes (μV) in 3 affected (ARSG) and 3 control ASTs. Individual datapoints are shown as dots.

**Figure 3:**
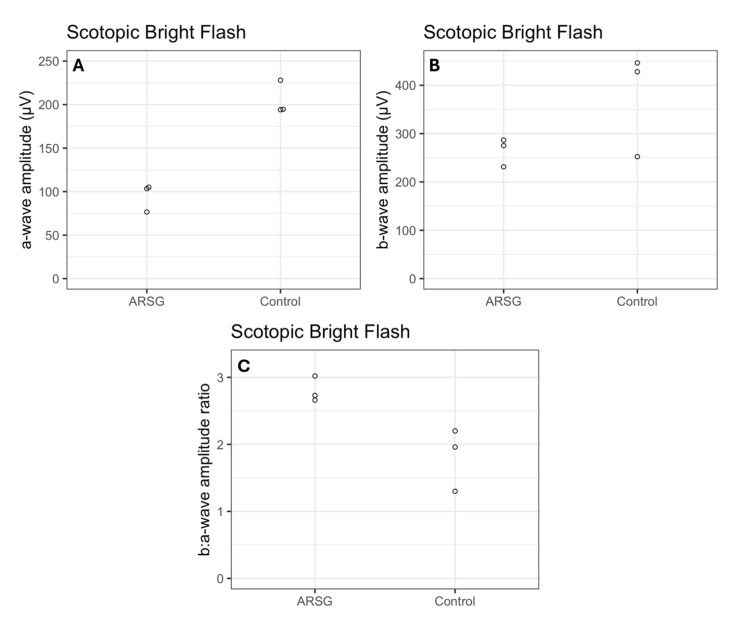
Mixed rod and cone responses assessed with scotopic bright flash a-wave amplitudes (μV) (**A**), b-wave amplitudes (μV) (**B**), and b:a-wave ratios (**C**) in 3 affected (ARSG) and 3 control ASTs. Individual datapoints are shown as dots.

**Figure 4:**
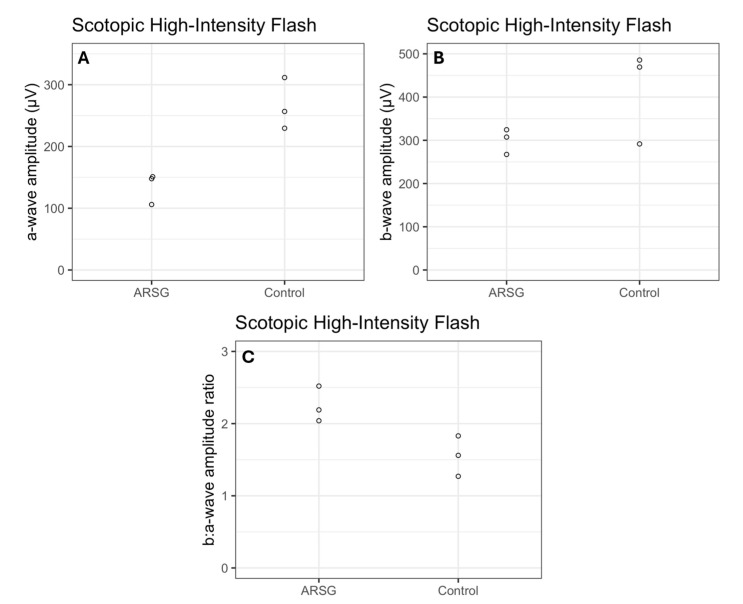
Mixed rod and cone function assessed with scotopic high-intensity flash a-wave amplitudes (μV) (**A**), b-wave amplitudes (μV) (**B**), and b:a-wave ratios (**C**) in 3 affected (ARSG) and 3 control ASTs. Individual datapoints are shown as dots.

**Figure 5:**
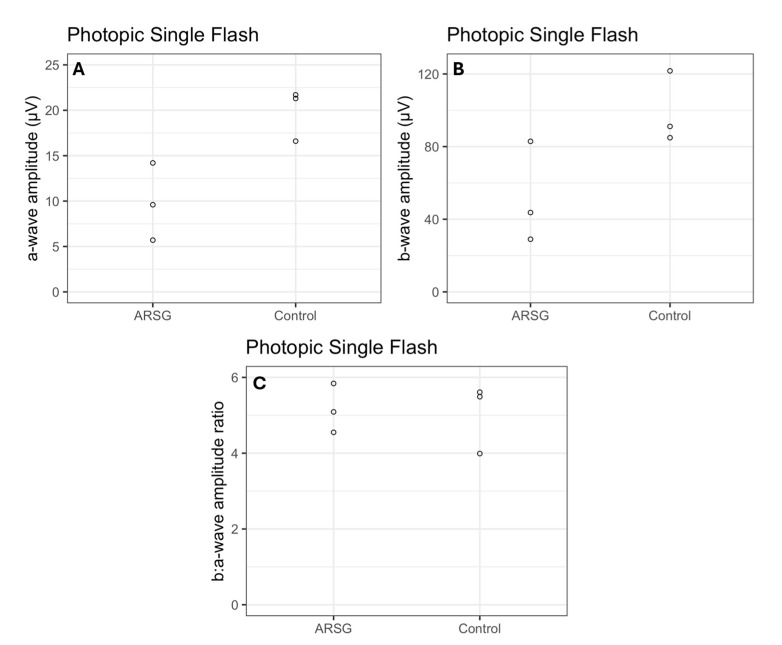
Cone responses as assessed with photopic single flash a-wave amplitudes (μV) (**A**), b-wave amplitudes (μV) (**B**), and b:a-wave ratios (**C**) in 3 affected (ARSG) and 3 control ASTs. Individual datapoints are shown as dots.

**Figure 6:**
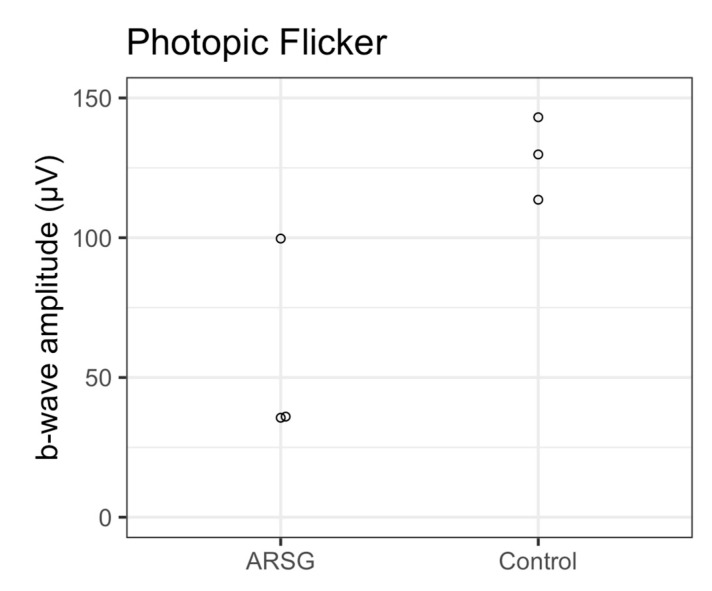
Cone responses as assessed with photopic flicker b-wave amplitudes (μV) in 3 affected (ARSG) and 3 control ASTs. Individual datapoints are shown as dots.

The affected dogs all exhibited substantially reduced ERG response amplitudes for both rods and cones relative to the unaffected dogs (Figure 1, Figure 2, Figure 3, Figure 4, Figure 5 and Figure 6). Pure rod response b-wave amplitudes from the fifth set of dim scotopic flashes, assessed after 20 min of dark adaptation, are shown in Figure 2: the amplitude was lower in all affected dogs compared to controls by an average of 57%. Scotopic “bright” and “high-intensity” flashes elicit a mixed rod–cone response. All bright scotopic mixed rod–cone a-wave amplitudes were lower in affected dogs than in controls by an average of 54% (Figure 3A). Not all b-wave amplitudes were lower in affected ASTs compared to controls, although the group average was 30% lower (Figure 3B). All b:a-wave ratios were higher in affected ASTs compared to controls by an average of 54% (Figure 3C). All high-intensity scotopic mixed rod–cone a-wave amplitudes were lower in affected dogs than in controls by an average of 49% (Figure 4A). Not all b-wave amplitudes were lower in affected ASTs compared to controls, although the group average was 28% lower (Figure 4B). All b:a-wave ratios were larger in affected ASTs compared to controls by an average of 45% (Figure 4C).

Photopic stimuli were presented after a 10 min period of light adaptation to elicit a pure cone response. All photopic single flash a-wave amplitudes were lower in affected ASTs compared to controls by an average of 51% (Figure 5A). All b-wave amplitudes were lower in affected ASTs compared to controls by an average of 48% (Figure 5B). The average photopic b:a-wave ratio differed by less than 3% between groups (Figure 5C). Photopic flicker b-wave amplitudes were lower in all affected ASTs compared to controls by an average of 56% (Figure 6).

Dog ARSG-4, who was 4 years of age at the time of assessment, did not exhibit neurological clinical signs and his ERG amplitudes showed no evidence of retinal dysfunction (Table 2).

## 7. Fluorescence Microscopy

Two ASTs homozygous for the *ARSG* risk allele were euthanized at 6.5 and approximately 7 years of age by their primary care veterinarians due to progressive ataxia. The dogs were sedated with acepromazine (~0.4 mg/kg im) followed by injection of pentobarbital (~150 mg/kg iv). Death was confirmed by absence of heartbeat and respiration and lack of a corneal reflex. An eye was enucleated from each dog after euthanasia, the cornea was removed, and the remainder of the eye was immersed in a solution consisting of 3.5% paraformaldehyde, 0.05% glutaraldehyde, 120 mM sodium cacodylate, and 1 mM CaCl_2_ at pH 7.4. After incubation in this solution for 5 days, an approximately 1 square cm region of the eyecup just superior to the optic nerve along the superior–inferior midline was dissected from the eye, incubated sequentially in 10% and 20% sucrose, embedded in Tissue Tek medium, and frozen on a block of dry ice. The retinas of these dogs were examined with fluorescence microscopy to assess whether they exhibited accumulation of autofluorescent storage material similar to that which occurs in other neural tissues of affected dogs. Cross sections of the tissue were cut at a thickness of 8 μm with a cryostat and mounted on Super Frost slides in 170 mM sodium cacodylate. Images of the unstained sections were obtained with a Zeiss Axiophot microscope using epi-illumination from a Prior Lemen light source, a 400 to 440 nm bandpass excitation filter, an FT 460 dichromatic beam splitter, and a 515 nm long-pass emission filter. Images were acquired with an Olympus DP 72 digital camera.

**Figure 7:**
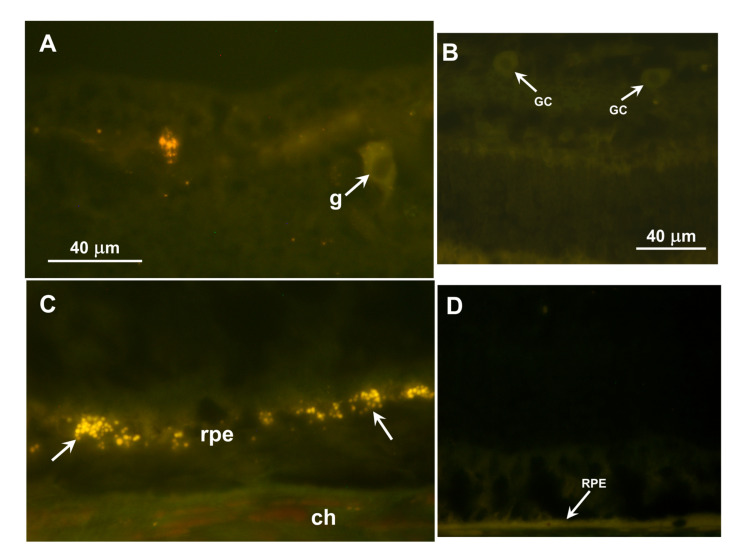
(**A**,**C**) Fluorescence micrographs of unstained cryostat sections of a retina from a 6.5-year-old AST who was euthanized due to progression of ataxia associated with homozygosity of the *ARSG* risk variant. (**A**) The inner retina including the ganglion cell layer adjacent to the vitreous (v). (**C**) The outer retina including the retinal pigment epithelium (rpe) and choroid (ch). Autofluorescent inclusion bodies (arrows) occurred primarily in the ganglion cell layer, but not in the ganglion cell bodies (g), and in the rpe. Scale bar in (**A**) indicates magnification of both micrographs. (**B**,**D**) Fluorescence micrographs of the same region of the inner (**B**) and outer (**D**) retina of a 5-year-old Australian Shepherd with a progressive neurological disorder of unknown etiology. The owners reported not observing any visual impairment in this dog. The retinal pigment epithelium (RPE) and ganglion cells (GCs), as well as the remainder of the retina, did not exhibit NCL-like autofluorescence. Bar in (**A**) indicates the magnification for (**A**,**C**); bar in (**B**) indicates the magnification for (**B**,**D**).

Inclusion bodies with autofluorescence properties typical of the NCLs were observed in the ganglion cell layers of the retinas and in the retinal pigment epithelium (RPE) of both dogs (Figure 7A,C). In both retinal layers, the inclusions were not uniformly distributed but occurred in clusters. The inclusions in the ganglion cell layer were not in the perinuclear zones of the ganglion cells as they are in some canine NCLs, but were in either the ganglion cell axons or Müller cell processes. None of these inclusions were present in the photoreceptor cell layer of the retina. No similar autofluorescent inclusions were present in the retina of a 5-year-old Australian Shepherd with an unrelated disorder that was not characterized by visual impairment (Figure 7B,D).

## 8. Discussion

The ERG data and owner observations suggest that retinal dysfunction and visual impairment occur in ASTs with the c.296G>A *ARSG* variant, consistent with reports of rod–cone dystrophy in human Usher patients with *ARSG* mutations and photoceptor degeneration in *arsg* knockout mice. Based on the assessments on a limited number of dogs, it appears that both rod and cone photoceptor function are impaired in the affected dogs. This is consistent with impaired photopic and scotopic visually mediated behavior reported by owners of many dogs homozygous for the *ARSG* risk variant. Of the affected dogs evaluated for this study, we were only able to obtain ECVO-standardized evaluations on 4 of 12 and on 3 of 18 control ASTs. The other dogs were scattered geographically and it was not practical to have them brought to our facility for evaluation. Most of these other dogs were evaluated by local ophthalmologists who were unable to obtain standardized ERGs with appropriate controls. The lack of implementation of standardized clinical ERG recording is an impediment to this type of study, and many cases of moderate retinal dysfunction in dogs are likely missed when the ECVO ERG protocols cannot be followed.

Confirmation of the ERG findings will require assessment of additional dogs using a standardized protocol capable of detecting moderate retinal functional impairment that includes appropriate controls. Based on the results of genetic testing for the *ARSG* risk variant in AST samples submitted to the University of Missouri Canine Genetics Laboratory, the risk allele appears to be relatively common in the breed. Therefore, veterinary ophthalmologists who can perform standardized ERG assessments should be able to evaluate additional affected dogs. Most ophthalmology practices are not equipped to perform ERG assessments compliant with the ECVO standard. However, the findings from this study suggest that assessing responses to low-intensity stimuli in dark-adapted dogs may be adequate to detect retinal functional impairment in this disorder as long as proper control data is available using the same protocols and instrumentation.

The ERG findings indicate the importance of standardized ERG assessments in evaluating retinal function in dogs. To accurately assess retinal function, it is important to employ uniform ERG methodology so that patient responses can be compared to normal responses obtained from healthy dogs using the same protocols and instrumentation. The standardized ERG protocol utilized in this study was first adopted by the European Conference on Veterinary Visual Electrophysiology in 2000 and updated in 2012 [20], but many veterinary ophthalmologists utilize a much more limited protocol that will only detect pronounced abnormalities in retinal function. Variations in ERG instrumentation also complicate comparisons of data obtained in different veterinary practices. Our findings illustrate the importance of systematic in-depth standardized ERG assessment in dogs in which potential retinal functional impairment is suspected.

Moderate visual deficits in dogs often go unrecognized by owners because dogs with such deficits can still navigate well in familiar surroundings, and progressively severe ataxia may make it difficult to detect impaired visually mediated behavior in the later stages of this disease (see Video S1). In the original report of the *ARSG*-related disorder in ASTs, it was stated that neither owners nor veterinarians reported visual impairment in any of the affected dogs that were evaluated [1]. That is in contrast to the present study in which 8 of 11 dogs homozygous for the risk variant were reported by their owners to have exhibited visual impairment. The basis for this apparent discrepancy is not evident, although there was no indication in the previous report that the owners and veterinarians were asked to comment specifically on visually mediated behavior. It is likely that visual impairment was not reported by owners and veterinarians in the original group of dogs due to focus on the more obvious signs of ataxia. Owner-reported assessments are subjective, but the consistency across owners, who were not aware that visual function was being studied when completing the questionnaires, suggests that changes to visual function do occur in at least some affected ASTs. Future studies could include more objective behavioral vision tests, such as maze navigation, optomotor response, or the cotton ball drop, to confirm the owner reports of visual impairment. This would require geographically dispersed evaluators to perform such tests in standardized formats. As with ERGs, there is a need for veterinary ophthalmologists to adopt standardized quantifiable visually mediated behavior assessments.

In the dogs that were evaluated for the current study, there were no consistent abnormalities in fundic appearances, despite consistent disease-related decreases in ERG amplitudes in the affected dogs relative to the unaffected controls. The kinetics of dark adaptation appeared to be altered in the affected dogs; peak scotopic ERG amplitudes were reached earlier in the affected dogs than in the control dogs. Since the peak scotopic ERG amplitudes also tended to be lower in affected dogs, this may simply indicate that there is less visual pigment in the retinas of the affected dogs to be regenerated during dark adaptation.

Because longitudinal ERG data were not obtained, this study did not establish whether retinal dysfunction is progressive, as is the case in human Usher patients. However, the finding that a young presymptomatic dog did not exhibit retinal dysfunction suggests that this may be the case. At 4 years of age, dog ARSG-4 showed no clinical signs of disease, and the ERG showed no indication of retinal dysfunction. In addition, owners of other affected dogs reported that the onset of apparent impairment in visually mediated behavior typically did not occur before 5 years of age and then gradually worsened over time. This is consistent with progressive deterioration in retinal function. Longitudinal studies over a 3-to-8-year age span may be necessary to document the age of onset and rate of progression of retinal dysfunction in this disease.

The owners of the affected dogs did not report signs of hearing impairment in their animals that occur in human subjects with *ARSG* mutations, but objective assessment of hearing was not performed. Affected dogs were reported to be hypersensitive to loud noises, which may be indicative of hearing impairment. Objective assessments of hearing in dogs can be performed using brainstem auditory evoked response testing. Such testing may detect hearing impairments in affected ASTs, particularly late in the disease progression.

The mechanism by which the defect in ARSG results in impaired retinal function remains to be elucidated. However, the finding that the RPE of affected dogs accumulated autofluorescent storage bodies suggests that disease-related RPE pathology may alter the RPE–photoreceptor cell interactions that are necessary for maintaining normal photoreceptor cell functions. ARSG is a lysosomal enzyme essential for the degradation of heparan sulfate, and heparan sulfate proteoglycans are present in the interphotoreceptor matrix [20]. ARSG expression in the dog RPE/choroid has been reported, consistent with this enzyme playing a role in RPE lysosomal function [21]. In healthy eyes, photoreceptor outer segment tip fragments and the associated interphotoreceptor matrix are phagocytosed by the RPE, and the resulting phagolysosomes are rapidly degraded by lysosomal enzymes [22,23,24]. Impaired RPE ARSG enzyme activity likely results in the accumulation of undegraded heparan sulfate proteoglycans and secondary accumulation of photoreceptor outer segment components in the RPE phagolysosomes. Accumulation of this storage material may impair RPE functions, including photoreceptor outer segment turnover and delivery of the visual pigment 11-cis retinal to the retina, which are necessary for normal photoreceptor cell function. This could lead to secondary impairment of photoreceptor function or survival. For example, RPE accumulation of lysosomal storage material may interfere with the ability of the RPE to metabolize and exchange retinoids with the photoreceptor cells, and the photoreceptor retinoid starvation could then cause photoreceptor degeneration [23,25,26,27,28,29,30,31,32,33,34,35,36]. Assessment of potential effects of ARSG deficiency on RPE cell function could be assessed with studies on RPE cells cultured from affected and control dog eyes or with mechanistic studies that could be performed with *arsg* mutant mice. The accumulation of autofluorescent storage bodies in the retinal ganglion cell layer suggests that glycosaminoglycan turnover also occurs normally in ganglion cells and/or Müller cells, but it is not known whether lysosomal storage body accumulation in these cells results in cell pathology. Autofluorescent storage body accumulation in the ganglion cell layer occurs in several canine NCLs and GM2 gangliosidosis, but in these cases the storage material is most abundant in the ganglion cell bodies [37,38,39,40,41].

While ERG response amplitudes were generally lower in the affected dogs relative to the unaffected controls, there was significant variability in these amplitudes among these dogs. This is consistent with the reported variability in age of onset and rate of disease progression in the neurological signs such as ataxia [1]. The basis for this variability is unknown, but it suggests that there may be genetic and/or environmental variables that modify the effect of ARSG deficiency. Identification of these modifiers could lead to strategies for therapeutic interventions.

The data presented here suggest that despite some differences in the phenotypes between human subjects, mice, and dogs with *ARSG*-related disorders, photoreceptor dysfunction is likely to be a consistent feature associated with *ARSG* variants in all three species in which these have been described. For both human and canine patients with late-onset visual deficits, variants in *ARSG* should be considered as potential causes.

## 9. Conclusions

Dogs that are homozygous for the *ARSG* variant responsible for a progressive neurological disorder appear to develop late-onset retinal dysfunction. Documentation of retinal functional impairment can be obtained with electroretinography, but only if the assessments are performed under standardized conditions that allow comparisons with unaffected dogs. To detect less than profound loss of retinal function, at a minimum ophthalmologists should standardize protocols internally and obtain control data from healthy dogs using the same protocols and instrumentation.

## Figures and Tables

**Table 1:** Summary of owner-reported vision in ASTs.

Genotype	Sex	Age (Years)	Ataxia	Photopic Vision	Scotopic Vision	Additional Assessments
**Homozygous for *ARSG* c.296G>A allele**	F	8	Severe	Normal	Normal	ERG (ARSG-1)
	M	8	Moderate	Mild	Moderate	ERG (ARSG-2)
	F	5.7	Moderate	Mild	Mild	ERG (ARSG-3)
	M	4	Normal	Normal	Normal	ERG (ARSG-4)
	M	3.3	Severe	Mild	Moderate	
	M	4	Moderate	Normal	Normal	Histology ^1^
	M	7.7	Mild	Normal	Mild	
	M	3+	Mild	Normal	Mild	
	F	4.2	Moderate	Normal	Normal	
	M	6.5	Moderate	Mild	Moderate	
	F	6.5	Moderate	Normal	Severe	Histology ^2^
	F	5.1	Severe	Normal	Moderate	
**Homozygous for WT *ARSG*** **Allele ^3^**	M	7	Normal	Normal	Normal	ERG (Control-1)
	F	2	Normal	Normal	Normal	ERG (Control-2)
	M	8	Normal	Normal	Normal	ERG (Control-3)

^1^ Owner report was submitted when the dog was 4 years of age. Euthanasia and tissue collection were performed when the dog was 6.5 years old. ^2^ Owner report was submitted when the dog was 6.5 years of age. Euthanasia and tissue collection were performed when the dog was 7 years old. ^3^ Owners of 15 additional ASTs homozygous for the WT allele for which ERG data were not obtained reported normal vision in their dog at 4 or more years of age under scotopic and photopic conditions.

**Table 2:** ERG results from dog ARSG-4, a 4-year-old AST homozygous for the c.296G>A allele who exhibited no neurological signs at the time of data collection.

Scotopic Dim Flash	b-wave	422.8 µV
Scotopic Bright Flash	a-wave	324.1 µV
	b-wave	648.4 µV
	b:a	2.00
Scotopic High-Intensity Flash	a-wave	400.1 µV
	b-wave	690 µV
	b:a	1.72
Photopic Single Flash	a-wave	22.2 µV
	b-wave	162.3 µV
	b:a	7.31
Photopic Flicker	b-wave	167.5 µV

a- and b-wave implicit times did not differ significantly between affected and control ASTs (Supplemental File S2).

## Data Availability

The raw data supporting the conclusions of this article will be made available by the authors on request.

## Supplementary Materials

The following supporting information can be downloaded at: https://www.mdpi.com/article/10.3390/vetsci12111078/s1, Supplemental File S1: Questionnaire, Supplemental File S2: Summary of implicit times.

## Abbreviations

The following abbreviations are used in this manuscript:

AST	American Staffordshire Terrier
*ARSG*	Arylsulfatase G
NCL	Neuronal Ceroid Lipofuscinosis
MPS	Mucopolysaccharidoses
ERG	Electroretinography
