# Supplementary material for: Retinal Function Deficits in American Staffordshire Terriers with a Late-Onset Neurodegenerative Disease Associated with an ARSG Variant
Source: Vet Sci. 2025 Nov 12;12(11):1078. doi: 10.3390/vetsci12111078 (PMC12656862; doi:10.3390/vetsci12111078)

Supplemental File S1

CANINE Neurological Diseases DNA Testing

Breed ___________________

Individual Dog Information

DCL # (we will complete):__________

Blood – Tissue – other ____________________

Registered Name ____ Call name ________________

Registration # ________________ Birth Date _____________ Sex? M – F Neutered/Spayed? Y – N

Sample Submission Date: ____________________ Color __________________________

Sample submitted for which DNA Test? ____

Owner: name ________________________ breeder's name ________________________

address ________________________ address ________________________

________________________ ________________________

phone (day) ______________________ phone ________________________

phone (eve) ______________________ e-mail ________________________

fax ________________________

e-mail ________________________

Does this dog exhibit any of the following conditions? (*Please attach history for any Yes answer*)

Y - N Allergies

Y - N Arthritis

Y - N Autoimmune Disorders

Y - N Bite or Tooth Abnormalities

Y - N Cancer / Tumors

Y - N Vision Problems

Y - N Deafness / Hearing Impaired

other (please list):

Y - N Digestive difficulties

Y - N Heart Problems

Y - N Hernia (where? ____________________)

Y - N Reproductive Problems

Y - N Seizures

Y - N Skin / Coat Problems

Y - N Skeletal Abnormalities (Hip Dysplasia, etc.)

Y - N Temperament Problems (shy, aggressive, etc.)

Testing done on this dog:

OFA/PennHip	Y - N	age at test: __________	result:________	#__________
CERF	Y - N	age last tested:_______	result:________	#__________
Thyroid	Y - N	age last tested:_______	result:________	

other (please list):

See following pages for questions on symptoms – please complete for ALL sampled dogs.

ATTACH PEDIGREE COPY TO THIS FORM

Please circle your response to the following;

- I am / am not willing to provide additional blood samples if needed for research.
- I will / will not consider donation of a tissue sample upon the death of this dog, and will discuss this decision with my veterinarian so that a notation is placed in my file.

I submit this sample and pedigree for the purpose of DNA research; I understand that the identity of dogs and owners participating in the research will not be revealed; and I have supplied complete and accurate information, to the best of my knowledge.

Signed: ______________________________________ date _________________

Canine Neurological Disease-specific Questionnaire

Has this dog been diagnosed as likely to be affected with a neurological disorder? Yes No

If yes, which disorder? ____

If yes, have any relatives of this dog been diagnosed with the same disorder? Yes No Don't Know

If yes, which relatives? Sire Dam Sibling Offspring Other ____________

Paternal Grandsire Paternal Grand-dam Maternal Grandsire Maternal Grand-dam

When is the best time to reach you by phone? _____________________________________

Veterinary Contact Information

Primary Care

Vet Name _________________________

Clinic Name _______________________

Address __________________________

City,St,Zip ________________________

Phone # __________________________

Email: ___________________________

Ophthalmologist

Name _____________________________

Clinic Name ________________________

Address ___________________________

City,St,Zip __________________________

Phone # ___________________________

Email: ____________________________

Neurologist

Vet Name _________________________

Clinic Name _______________________

Address __________________________

City,St,Zip ________________________

Phone # __________________________

Email: ___________________________

Other Specialist

Name _____________________________

Clinic Name ________________________

Address ___________________________

City,St,Zip __________________________

Phone # ___________________________

Email: ____________________________

May we have your permission to contact your veterinarians to request records and discuss your dog's health history, diagnostic testing, and possible treatment options? Yes No

Signed: ____________________________________ date: ________________

Behavior and Activity survey follows – please complete for all sampled dogs

CHANGES IN *BEHAVIOR*

Compare this dog's current behavior to its earlier behavior. Please circle the correct answer.
If you need additional space to describe changes, please use back of form or attach additional pages.

	**Normal** - or - **Degree of Change**				**Describe Changes and Indicate <u>Age of Onset</u>**
1. Housetraining	normal	mild	moderate	severe	________________
2. Interest in food (eating habits)	normal	mild	moderate	severe	________________
3. Appears nervous	normal	mild	moderate	severe	________________
4. Interaction/socialization with other dogs	normal	mild	moderate	severe	________________
5. Aggressiveness to other dogs	normal	mild	moderate	severe	________________
6. Aggressiveness to people	normal	mild	moderate	severe	________________
7. Tolerance to grooming or bathing	normal	mild	moderate	severe	________________
8. Tolerance to being alone	normal	mild	moderate	severe	________________
9. Ability to recognize/respond to commands	normal	mild	moderate	severe	________________
10. Ability to recognize or respond to name	normal	mild	moderate	severe	________________
11. Recognizes you or other familiar people	normal	mild	moderate	severe	________________
13. Responses to noise/loud sounds	normal	mild	moderate	severe	________________
14. Development of compulsive behavior	normal	mild	moderate	severe	________________
15. Circling	normal	mild	moderate	severe	________________
16. Wakes you more at night	normal	mild	moderate	severe	________________
17. Inappropriate or persistent vocalization	normal	mild	moderate	severe	________________

CHANGES IN *PHYSICAL ACTIVITY*

Compare this dog's current physical activity to its earlier activity and ability. Please circle the correct answer.
If you need additional space to describe changes, please use back of form or attach additional pages.

	**Normal** - or - **Degree of Change**				**Describe Changes and Indicate <u>Age of Onset</u>**
18. Climbing up or down stairs	normal	mild	moderate	severe	______________________________
19. Tremors or shaking	normal	mild	moderate	severe	______________________________
20. Seizures	normal	mild	moderate	severe	______________________________
21. Increased stiffness or weakness	normal	mild	moderate	severe	______________________________
22. Difficulty in movement or coordination	normal	mild	moderate	severe	______________________________
23. Changes in posture ("roached" back)	normal	mild	moderate	severe	______________________________
24. Tail carriage when alert & interested	normal	mild	moderate	severe	______________________________
25. Ability to see during the day	normal	mild	moderate	severe	______________________________
26. Ability to see at night in dim light	normal	mild	moderate	severe	______________________________
27. Head movements	normal	mild	moderate	severe	______________________________
28. Trance-like behavior	normal	mild	moderate	severe	______________________________
29. Bumps into objects, clumsy	normal	mild	moderate	severe	______________________________

Please describe any other health problems or behavioral abnormalities:

____

____

Supplemental File S2 ERG Implicit Times

Table S1. Summary of implicit times.

		ARSG Mean	Control Mean
Scotopic Dim Flash	b-wave	69.4	74.8
Scotopic Bright Flash	a-wave	14.7	13.0
	b-wave	38.8	35.0
Scotopic High-Intensity Flash	a-wave	13.9	11.1
	b-wave	42.1	36.1
Photopic Single Flash	a-wave	10.6	10.5
	b-wave	23.9	23.9
Photopic Flicker	b-wave	22.6	22.8

Figure S1. ERG implicit times in 3 affected and 3 control ASTs.

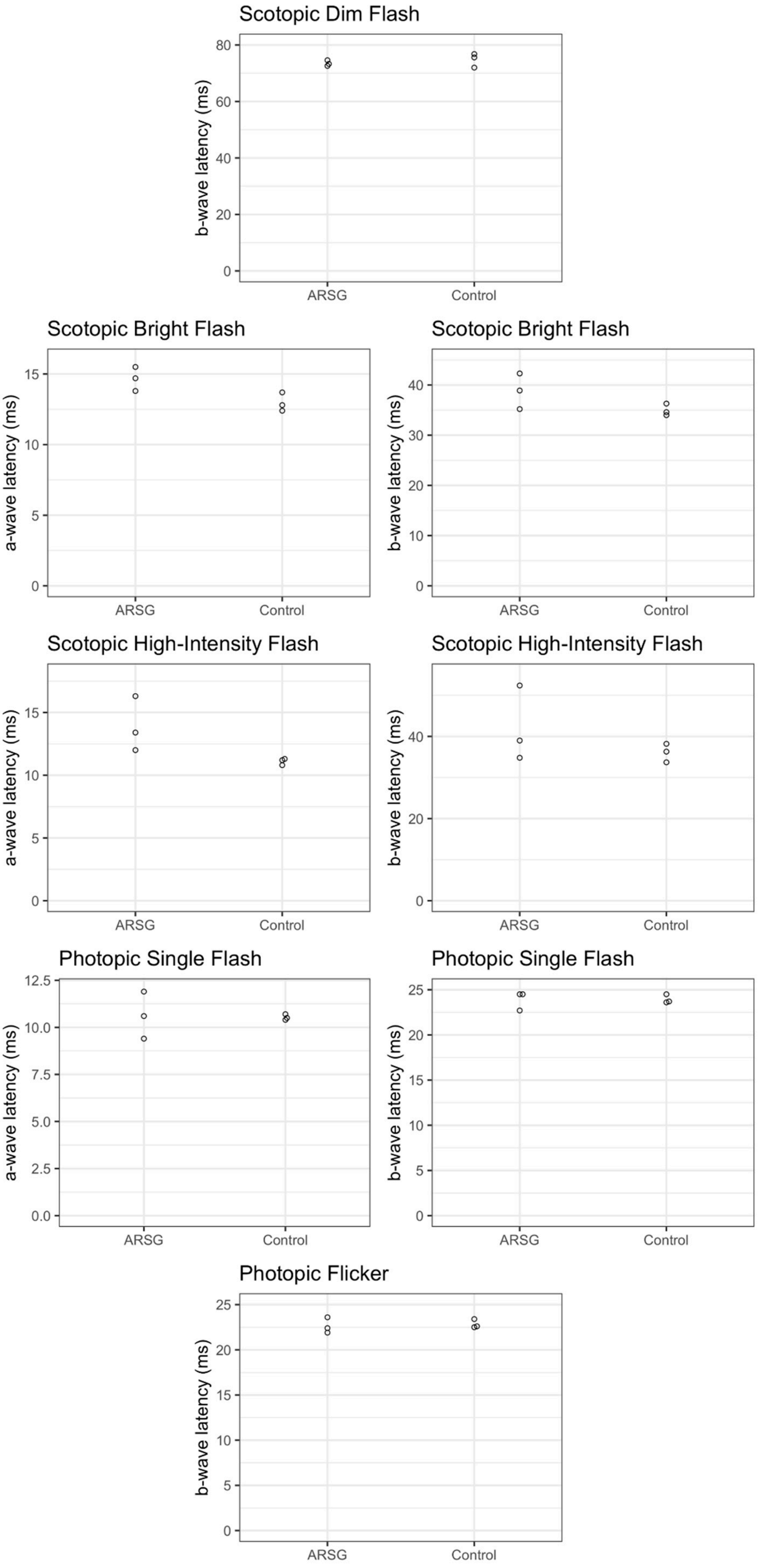

Supplement: Supplementary file 1 [file vetsci-12-01078-s001.zip › ARSG Manuscript_Supplemental Files Combined.pdf]
